# Prognosis of patients with early salvage radiotherapy and low persisting or increasing PSA levels after radical prostatectomy for prostate cancer. A retrospective comparative analysis

**DOI:** 10.1016/j.ctro.2026.101121

**Published:** 2026-02-11

**Authors:** Reinhard Thamm, Sophia Scharl, Luca Gartner, Dirk Heinz Gerhard Böhmer, Alessandra Siegmann, Daniel Zips, Cornelia Horsch, Benjamin Mayer, Thomas Wiegel

**Affiliations:** a University Hospital Ulm, Clinic for Radiotherapy and Radiooncology, Albert-Einstein-Allee 23, 89081 Ulm, Germany; b Charité - Universitätsmedizin Berlin, Corporate member of Freie Universität Berlin and Humboldt-Universität zu Berlin, Klinik für Radioonkologie und Strahlentherapie, Hindenburgdamm 30, 12203 Berlin, Germany; c Ulm University, Institute for Epidemiology and Medical Biometry, Schwabstraße 13, 89075 Ulm, Germany

**Keywords:** Prostatic neoplasms, Radiotherapy, Salvage therapy, Prostate-specific antigen

## Abstract

**Title:**

Prognosis of patients with early salvage radiotherapy and low persisting or increasing PSA levels after radical prostatectomy for prostate cancer. A retrospective comparative analysis.

**Introduction:**

European prostate cancer guidelines include a “weak” recommendation to add hormonal therapy (ADT) to salvage radiotherapy (SRT) for better prognosis with persistent PSA values after radical prostatectomy (RP). In this study, prognosis with persistent PSA versus a PSA increase above undetectable after RP was compared.

**Methods:**

In this retrospective study of patients who underwent SRT to the prostate bed without ADT (pre-SRT-PSA ≤ 0.3 ng/ml), 243 patients had a PSA increase from undetectable (post-op PSA ≤ 0.05 ng/ml), and 110 patients had persisting PSA after RP (post-op PSA > 0.05 ng/ml). Primary endpoints: clinical progression-free survival (PFS), biochemical PFS (BPFS), metastasis-free survival (MFS), overall survival (OS). Results: BPFS rates were 66.4% with low persistent PSA versus 71.6% with recurrent PSA (p = 0.253; median follow-up, 60 [25–92] months). The low-persistent and recurrent groups also did not differ for MFS, PFS, or OS (all p > 0.05). In univariate and multivariate analyses, T3-4 and Gleason grade 4-5 predicted BPFS; T-status, R-status (R1,2), and Gleason grade influenced PFS; Gleason grade predicted MFS; and T-status predicted OS. In multivariate analysis, PSA persistence did not predict any survival outcomes (all p > 0.05).

**Conclusion:**

Prognosis with low persistent PSA values seems comparable to that with PSA levels increasing above undetectable. In the absence of other factors, ADT could be omitted in this favorable patient group. Prospective trials are needed.

## Highlights

• Prognosis is favorable with low persistent PSA and low pre-SRT PSA after RP for PCa.
• In patients with low persistent PSA, antihormonal therapy may be omitted.
• ADT may be initiated later after SRT if PSA rises or does not become undetectable.

## Introduction

Radical prostatectomy (RP) is a primary treatment option for localized prostate cancer (PCa). In the follow-up care of patients with PCa, PSA level is used for monitoring the continued absence of tumors. The first postoperative PSA level is usually measured at 6–12 weeks, depending on the preoperative PSA value [1], [2].

In 8% to 42% of cases [1], [3], [4], tumor cell presence from persistent local disease or pre-existing metastases must be suspected because the PSA level remains measurable at ≥ 0.1 ng/ml. This cutoff value is commonly accepted as defining PSA persistence [1]. Progression-free survival (PFS) is reported to be shortened and overall survival (OS) affected in these patients [1], [3], [5], [6], highlighting the need for risk-adapted therapies, such as salvage radiotherapy (SRT) with or without hormone therapy (ADT).

About 28% of patients experience a later PSA increase after having an undetectable value post-prostatectomy [7]. These patients are at risk for developing metastatic disease within 10 years, resulting in a shorter survival time. SRT with or without additional hormone therapy can prevent further progression and currently is the only curative therapeutic approach.

With PSA persistence or PSA progression, the PSA value before SRT is considered a prognostic marker [8], [9]. Accordingly, patients with very low PSA values, regardless of persistence or recurrence, are expected to have a more favorable prognosis than those with higher pre-SRT values [10]. In the current era of ultrasensitive PSA testing, it is possible to identify the patient groups that may benefit from early SRT.

In this retrospective study, outcomes for patients with low pre-SRT PSA levels were examined. Patients with low PSA persistence were compared those with PSA progression, with a focus on low pre-SRT PSA levels of ≤ 0.3 ng/ml.

## Methods

This study was based on a previously published retrospective cohort [8] of 698 patients from two high-volume radiation oncology centers (Charité Berlin, Campus Benjamin Franklin, University Hospital of Ulm). All patients had received SRT of the prostate bed without concomitant anti-hormonal therapy between 1997 and 2018. Exclusion criteria were lymph node or distant metastases based on postoperative or pre-SRT information.

Following exclusion, 353 patients were included who had pre-SRT PSA values ≤ 0.3 ng/ml. All 353 patients received SRT to the prostatic fossa, with or without the bed of the seminal vesicles. The median dose was 70.2 Gy (95% confidence interval, 70.2–72 Gy; range, 59.4–74 Gy). All patients underwent 3D-planned radiation therapy, and 42.1% underwent intensity-modulated therapy, volumetric-modulated radiotherapy, or a mixed technique for the boost volume. The planning target volume covered the prostate bed with a safety margin of 0.7–1 cm. The seminal vesicle bed was included in the case of pT3-4. Among the overall patient group, 13.8% underwent PET-CT before SRT, which had been increasingly implemented in radiotherapy planning after 2004.

For selecting a favorable patient cohort, we defined a postoperative PSA nadir > 0.05 ng/ml as PSA persistence, which described 110 of these cases. This threshold value of 0.05 ng/ml was individually chosen because the detection limits of modern PSA test methods are already at 0.01 ng/ml and support refinement of prognosis [11]. The remaining 243 patients experienced PSA progression from an undetectable postoperative PSA nadir of ≤ 0.05 ng/ml to a maximum pre-SRT PSA value of 0.3 ng/ml. Patient characteristics are listed in Table 1.

**Table 1:** Definitions and descriptive characteristics of included patients.

**Patient group**		**Recurrent PSA** **n = 243**	**Persisting PSA** **n = 110**	
**Definition** Post-op PSA nadir (resulting range) Pre-SRT PSA		≤0.05 ng/ml (0.0–0.05 ng/ml) ≤0.3 ng/ml	>0.05 ng/ml (0.051–1.3 ng/ml) ≤0.3 ng/ml	
**Variable**	**Category**			**P**
**Age at SRT** years, median (IQR)		66 (61–70)	65 (61–69)	**0.027**
**Pre-op PSA** ng/ml, median (IQR)		8.45 (5.71–11.55)	9.70 (8.79–11.08)	**0.004**
**Tumor stage/ISUP**	T1-2 T3a, b, T4	128 (54.7%) 115 (47.3%)	52 (47.3%) 58 (52.7%)	0.348
**Gleason Grade/ISUP**	1–3 4–5 Unknown	196 (80.7%) 46 (18.9%) 1 (0.4%)	85 (77.3%) 24 (21.8%) 1 (0.9%)	0.510
**Surgical margins**	R0 R1/2 Unknown	107 (44.0%) 117 (48.1%) 19 (7.8%)	54 (49.1%) 49 (44.5%) 7 (6.4%)	0.434
**Time from RP to SRT** median (IQR) value (%)	Months	23 (12–44)	12 (4–33)	**0.013**
	≤1 year >1 year	62 (25.5%) 181 (74.5%)	56 (50.9%) 54 (49.1%)	**< 0.001**
**Pre-SRT PSA** ng/ml, median (IQR)		0.16 (0.09–0.22)	0.19 (0.13–0.25)	**< 0.001**
**Radiotherapy dose** Gy, median (IQR)		70.2 (66.6–72)	72 (70.2–72)	0.170
**Post-SRT PSA-Nadir** ng/ml, median (IQR)		0.02 (0.0–0.05)	0.04 (0.0–0.07)	**< 0.001**
	≤0.1 ng/ml >0.1 ng/ml	216 (88.9%) 27 (11.1%)	91 (82.7%) 19 (17.3%)	0.112
**Follow-up** months, median (IQR)		60 (27–90)	59.5 (25–99)	0.571
Significant p values are indicated in boldface. Percentages refer to the proportion in the recurrent PSA/persistent PSA group.				

Biochemical recurrence (BCR) after SRT was defined as two rising PSA values of 0.2 ng/ml above the post-SRT nadir or the start of ADT. Biochemical PFS (BPFS) was defined as the time between the end of SRT and the date of BCR. Clinical recurrence (CR) was defined as clinical evidence of PCa or death from any cause, and PFS was defined as the time interval from the end of SRT to CR. Metastasis-free survival (MFS) and OS were defined respectively as the time intervals from the end of SRT to the detection of metastases or death for any reason. When the PSA laboratory value was below the threshold value, the threshold value was used for the calculation.

Continuous data are presented as medians and interquartile ranges (IQR, 25th to 75th percentiles), and absolute frequencies and relative percentages were used for categorical data. Statistical comparisons were performed using the *t*-test or Mann-Whitney *U* test for continuous data, respectively, or the chi-square test for the comparison of categorical attributes. Kaplan-Meier curves and log-rank tests were used to analyze survival outcome parameters. The impact of disease- and treatment-related parameters was evaluated using Cox regression analysis. Data were analyzed with MedCalc® Statistical Software version 23.1.7 (MedCalc Software Ltd, Ostend, Belgium; https://www.medcalc.org; 2025) and the R Project for Statistical Computing (version 4.5.0, https://www.r-project.org/). A significance level of p < 0.05 was assumed for all analyses. Data collection was approved by the Ethics Committee of the University Hospital Ulm (number 391/15).

## Results

The median follow-up time for the entire cohort of 353 patients after SRT was 60 months (IQR, 25–91.5 months). The patient group with PSA persistence showed only minor differences in characteristics compared to the group with PSA recurrence. Criteria such as age, tumor stage, Gleason grade, and positive resection margins did not differ significantly between the two groups (Table 1). Those with PSA persistence had a slightly higher preoperative PSA value and had been referred to SRT earlier, with higher pre-SRT PSA values; however, they received comparable median irradiation doses (Table 1). Before 2007, a significantly lower median radiation dose was selected (66.6 Gy, IQR 66.6–70.2) compared to after that year (72 Gy, IQR 72.0–72.0). This difference was statistically equally distributed between the two patient groups (Table 2). After SRT, 87.0% of all patients experienced a PSA decline to ≤ 0.1 ng/ml, with a median time to this threshold of 6 months (IQR, 2–12 months). The groups did not differ in time to decline to the PSA nadir (median 6 months with persistence vs. 5 months with recurrence, p = 0.756).

**Table 2:** Univariate (UV) and multivariate (MV) analyses of PSA persistence, T-status, R-status, and Gleason grading for BPFS, PFS, MFS, and OS.

**Variables**	**Levels**		**Biochemical progression-free survival (BPFS)**			**Clinical progression-free survival (PFS)**	
			**HR (95% CI)**	**P**		**HR (95% CI)**	**P**
Post-op PSA, patient group	Persisting vs.recurrence	**UV**	1.29 (0.83–1.99)	0.225		1.48 (0.93–2.35)	0.101
		**MV**	1.17 (0.75–1.81)	0.486		1.13 (0.69–1.85)	0.641
T-status	T3a, b, T4 vs.T1,2	**UV**	2.75 (1.74–4.34)	**<0.001**		2.20 (1.35–3.58)	**0.001**
		**MV**	2.35 (1.47–3.77)	**<0.001**		1.91 (1.13–3.22)	**0.015**
R-status	R1, 2 vs.R0	**UV**	0.76 (0.49–1.16)	0.120		0.66 (0.41–1.06)	0.085
		**MV**	n.a.^a^	n.a.^a^		0.61 (0.38–0.99)	**0.046**
Gleason grading	GG 4, 5 vs.GG 1, 2, 3	**UV**	2.61 (1.67–4.07)	**<0.001**		2.92 (1.81–4.70)	**<0.001**
		**MV**	2.04 (1.28–3.23)	**0.003**		2.72 (1.65–4.50)	**<0.001**
**Variables**	**Levels**		**Metastasis-free survival (MFS)**			**Overall survival (OS)**	
			**HR (95% CI)**	**P**		**HR (95% CI)**	**P**
Post-op PSA, patient group	Persisting vs.recurrence	**UV**	0.47 (0.10–2.17)	0.331		0.71 (0.25–1.97)	0.507
		**MV**	0.39 (0.08–1.85)	0.237		0.56 (0.02–1.59)	0.278
T-status	T3a, b, T4 vs.T1,2	**UV**	0.53 (0.16–1.82)	0.314		3.20 (1.06–9.67)	**0.039**
		**MV**	n.a.^a^	n.a.^a^		3.52 (1.16–10.71)	**0.027**
R-status	R1, 2 vs.R0	**UV**	0.74 (0.23–2.44)	0.624		1.24 (0.50–3.10)	0.642
		**MV**	n.a.^a^	n.a.^a^		n.a.^a^	n.a.^a^
Gleason grading	GG 4, 5 vs.GG 1, 2, 3	**UV**	3.86 (1.17–12.68)	**0.026**		1.87 (0.67–5.19)	0.233
		**MV**	4.27 (1.28–14.23)	**0.018**		n.a.^a^	n.a.^a^

HR, hazard ratio (>1 indicates increased risk); CI, confidence interval; GG, Gleason grading.

a The multivariate model included all variables from univariate models with p < 0.1. Significant p values are indicated in boldface.

Oncological outcome parameters (2- and 5-year BPFS, PFS, MS, and OS) did not differ significantly between the two groups (Table 3), with only a trend toward worse PFS in patients with persistent PSA levels (p = 0.099). The development of metastases was very low in both groups, and OS rates were high (Table 3).

**Table 3:** Oncological outcome parameters at 2 and 5 years after SRT, Kaplan–Meier estimation.

**Variables**	**Recurrent PSA** **n = 243 (68.8%)**			**Persisting PSA** **n = 110 (31.1%)**			
	**2-year [%]**	**5-year [%]**		**2-year [%]**	**5-year [%]**		**P**
**Biochemical progression-free survival (BPFS)**	90.4	71.6		77.5	66.4		0.253
**Clinical progression-free survival (PFS)**	93.2	79.4		88.8	72.0		0.099
**Metastasis-free survival (MFS)**	98.5	96.6		100	98.8		0.319
**Overall survival (OS)**	99.5	97.5		100	96.9		0.505

Univariate and multivariate analyses showed no statistically significant effect of group membership on BPFS, PFS, MS, or OS. Higher T category and Gleason grade were associated with a significantly higher risk of biochemical or clinical progression. MFS was influenced mainly by higher Gleason grade, whereas OS was influenced primarily by a higher T category. Residual tumor (R1/2) had a statistically borderline effect on clinical progression but no significant impact on other oncologic outcome parameters (Table 2).

## Discussion

In this retrospective cohort study, we found that prognosis was similarly favorable between patients with persisting PSA values and patients with PSA levels increasing above undetectable (pre-SRT PSA ≤ 0.3 ng/ml).

A worse oncologic prognosis has been reported for patients with persistent PSA levels [1], [4], [6], [12], [13] after RP for PCa compared to those with non-detectable PSA levels. For patients with persistence, SRT is the first option to prevent further progression, and the effectiveness of SRT has been documented in many studies [1], [3], [4], [14]. PSA level before SRT also has been identified as a biomarker of biochemical progression [13], [15] and risk of oncological progression [13], [15], [16]. Most studies of persistent PSA levels, however, have involved heterogeneous groups of patients (with persistent or recurrent PSA and with varying PSA levels) who underwent SRT, while our study focuses on selected patients with low PSA levels.

Preisser et al. [4] published findings from a retrospective study of 11,604 patients with persistent PSA (8.8%, n = 1025) or PSA recurrence (91.2%). Fifteen years after RP, with a median follow-up of 46.4 months, MFS and OS were 53.0% and 93.2% (p < 0.001) for patients with persistent PSA and 64.7% and 81.2% (p < 0.001) for patients with undetectable PSA. These authors concluded that SRT could improve OS and cancer-specific survival in patients with persistent PSA, but no subgroup analysis was performed in patients with lower persistent PSA and lower pre-SRT PSA levels.

Tilki et al**.** [17] published findings of an analysis of 25,551 retrospective patients with SRT for PSA progression after RP. After a median follow-up of 6 years, the adjusted hazard ratio for all-cause mortality became significant when pre-SRT PSA level rose above a cutoff of 0.25 ng/ml, particularly in patients with high-risk characteristics (T3/4 or post-RP Gleason score 8–10). These findings hinted that a low pre-SRT PSA level might be associated with a better prognosis, as also suggested in an earlier and smaller mixed cohort study of patients treated with SRT for a detectable PSA after RP [18]. With a median follow-up of 5 years, the 5-year freedom from biochemical failure rates in that study were 71%, 63%, 54%, 43%, and 37% in subgroups with pre-SRT PSA levels of 0.01–0.2, 0.21–0.5, 0.51–1.0, 1.01–2, and > 2 ng/ml, respectively. Comparable data have been reported in other studies [19], [20].

Overall, these findings point to a better prognosis for patients with low PSA after RP, regardless of whether they had persistent PSA or PSA progression. For these reasons, here we focused on patients with low pre-SRT PSA values (≤ 0.3 ng/ml) classified as having PSA persistence or progression based on a post-RP nadir of 0.05 ng/ml. To the best of our knowledge, no comparable studies have been conducted exclusively on this favorable patient group.

Whether supplementary hormone therapy should be used also needs to be addressed. Adding ADT to SRT might improve survival probability, but the indication to administer ADT in patients with persistent PSA is not standardized because of a lack of prospective randomized controlled trials [12], [14], [21], [22]. Therefore, the European Association of Urology guidelines include a “weak” recommendation to add ADT to SRT. This recommendation is based on retrospective studies of the impact of hormone therapy on improving oncological prognosis.

The phase 2 GETUG-AFU 22 study [23], [24], conducted in France, focused on patients with persistent PSA levels to clarify the need for additional hormone therapy and assess its toxicity following SRT. To date, this study has been published in abstracts, but not in a peer-reviewed journal.

In total, 64 patients who received immediate SRT alone and 61 patients who received SRT plus 6 months of ADT were evaluated. Persistent PSA was defined as a post-RP PSA level ≥ 0.2 ng/ml. The median post-RP PSA level in this cohort was 0.6 ng/ml (range 0.12–3.65 ng/ml). After a median 75 months, there was an improvement in 5-year MFS in patients receiving immediate SRT and ADT (hazard ratio, 0.51; p = 0.048), but the addition of ADT did not significantly improve event-free survival or biochemical progression. In fact, the median post-RP PSA in that study was almost threefold higher than in our cohort (0.19 ng/ml; Table 1), and the observations in the GETUG-AFU 22 cohort may be attributable to a higher risk for progression. This study does not support the fact that additional ADT may be necessary in patients with low PSA levels but may indicate that hormone therapy can be avoided.

In a post-hoc analysis of the RTOG 9601 phase III trial data by Sood et al. [25], patients (n = 760) who received SRT (64.8 Gy) with or without antiandrogen therapy for biochemical persistence (nadir PSA > 0.4 ng/ml, n = 90) had a 2.5 times higher risk of experiencing local or metastatic failure than patients with BCR (nadir PSA ≤ 0.4 ng/ml, n = 670). Again, one possible reason for the worse oncological outcomes in this cohort might have been the high PSA nadir cutoff of 0.4 ng/ml. The prospective data collected in GETUG-AFU 22 and those of the post hoc analysis of the RTOG 9601 data correspond well. This agreement supports the hypothesis that hormone therapy is necessary in higher risk cases, as indicated by higher post-RP PSA levels. In contrast, the pre-SRT PSA levels in our cohort were significantly lower than in these previous studies. The current comparatively good oncological results could indicate that in a highly selected patient population, SRT alone may be sufficient and hormone therapy may be necessary.

Burdett et al. [26] (the DADSPORT Collaboration) conducted a *meta*-analysis of aggregated data from five completed randomized controlled trials on postoperative radiotherapy (adjuvant and mostly salvage radiotherapy; six trial comparisons: RADICALS-HD, RTOG 9601, RTOG 0534, GETUG-AFU 16, and –22) and analyzed the role of hormone therapy in 4411 participants. The authors concluded that ADT slightly improves MFS and prostate cancer–specific survival, with 4% improvements for both outcomes at 8 years. The extent of improvement in OS is small and may be limited to people with higher risk factors. In the aggregated data analysis, the effect of ADT on MFS and OS was statistically lowest with pre-RT PSA ≤ 0.3 ng/ml, which agrees with the current findings.

In our retrospective patient group, most patients (87%) had not undergone prostate-specific membrane antigen (PSMA)-PET-CT to detect local residual tumors or rule out locoregional or distant metastases prior to SRT. Most studies on this topic have not involved routine PSMA-PET-CT examination prior to SRT, but the question arises as to whether patients with very low PSA levels could benefit from this modern diagnostic method. In fact, this examination method describes current tumor extent in approximately 54%–67% of patients, even with PSA below 0.5–0.6 ng/ml [27], [28]. In a recent retrospective analysis of 115 patients with BCR after RP and very low PSA values of 0.2 ng/ml before PSMA-PET-CT, this examination appeared to be useful, especially in cases of PSA doubling time ≤ 12 months or Gleason score ≥ 7b [29]. Therefore, PSMA-PET-CTs seem justifiable, especially when risk factors are present, even with low pre-SRT PSA values. Patients thus can be divided into low- and high-risk groups, and with this risk classification, PSMA-PET–planned SRT may predict outcomes [30], [31].

Taken together, our results indicate that patients with low persistent PSA have a prognosis that is as good as those with PSA recurrence when SRT is administered in patients with low pre-SRT PSA values. The use of ADT in addition to SRT does not appear to be necessary and may be initiated later if PSA does not fall to undetectable levels or rises again after SRT. This inference is supported by findings in another analysis by Scharl et al. showing that a post-SRT PSA value < 0.1 ng/ml is a significant predictor of favorable BPFS and MFS [8].

Our study has some limitations. With the retrospective data collection, standardized PSA control within 6–8 weeks after RP could not be guaranteed. The number of patients with persistent PSA values (n = 110) was relatively low and the risk factors were not distributed equally between the groups, which may limit the significance of these results. Furthermore, because of the long-time frame for data collection, treatment procedures in the cohort did not always reflect current standards of care, such as target volume definition and intensity modulated therapy.

## Conclusion

To the best of our knowledge, this is the first evaluation to show that patients with low persisting PSA values (post-RP PSA > 0.05 ng/ml) seem to have a prognosis that is comparable to that of patients with PSA levels increasing above undetectable when the pre-SRT PSA was ≤ 0.3 ng/ml. Hormone therapy can probably be avoided in this patient group, and PSMA-PET-CT may be useful in patients with risk factors. Due to the weaknesses inherent to a retrospective analysis, the results should be interpreted with caution but are nevertheless useful for generating hypotheses. Further studies are required to validate these findings.

## Declaration of competing interest

The authors declare that they have no known competing financial interests or personal relationships that could have appeared to influence the work reported in this paper.
